# Arsenic content and exposure in brown rice compared to white rice in the United States

**DOI:** 10.1111/risa.70008

**Published:** 2025-02-28

**Authors:** Christian Kelly Scott, Felicia Wu

**Affiliations:** ^1^ Department of Food Science and Human Nutrition Michigan State University East Lansing Michigan USA; ^2^ Department of Agricultural, Food, and Resource Economics Michigan State University East Lansing Michigan USA

**Keywords:** arsenic, brown rice, inorganic arsenic, rice, white rice

## Abstract

Brown rice is often considered a healthy alternative to white rice due to the additional nutrients contained within the rice bran. However, the proposition of improved health outcomes by replacing white rice with brown rice in diets ignores a potential food safety concern: arsenic exposure. In this manuscript, we seek to critically compare potential arsenic exposure and the associated risks between brown and white rice for US populations. Rice bran and brown rice are shown to have a higher arsenic content and inorganic arsenic concentration than the grain endosperm or white rice. Americans who regularly consume brown rice versus white rice were found to have higher estimated arsenic exposures. Because young children consume considerably more food relative to their bodyweights than adults, brown rice consumption in young children was found to more substantially increase foodborne arsenic exposures. However, there are no acute public health risks indicated for the general American population from rice‐related arsenic exposures. Risk–benefit analyses are needed to assess relative risks of arsenic exposure in brown rice compared with the nutritional benefits, in comparison to white rice.

## 1 INTRODUCTION

Rice is a global dietary staple that provides essential calories to billions of people around the world (Fukagawa & Ziska, 2019). In the United States, rice consumption has continually increased per capita on average in the last 50 years (Batres‐Marquez et al., 2009). In that time, it has been a common assumption that brown rice is more healthful than white rice (Selvam et al., 2017; Wu et al., 2018), because of higher micronutrient and fiber content (Fukagawa & Ziska, 2019; Sun et al., 2010). Although brown rice and white rice come from the same rice species, brown rice is relatively less processed, which allows for some bran and germ content, resulting in a brown appearance. White rice, in contrast, is refined to strip the bran and germ, leaving the white endosperm for consumption (Wu et al., 2023). The milling and processing of rice can result in visually contrasting grain that results in not just differences in appearance and nutritional content, but also differences in public perception, price, cooking methods, culinary characteristics, taste, and toxic element content. It is this last difference that our article explores.

For some, the expected nutritional benefits of brown rice have made it more desirable (Mir et al., 2016, 2020). For others, factors such as taste, cultural preferences, cooking time, price, and limited storage life make brown rice less preferred than white rice (Kumar et al., 2011; Mohan et al., 2017). Brown rice retains much of the grain's nutrients found within the bran and the germ, including fiber, protein, niacin, and folate, with potential benefits to consumers. However, the bran and germ of brown rice also retain higher concentrations of certain harmful toxic elements such as arsenic, which are taken up from the soil during its cultivation (Feng Ma et al., 2008; Meharg & Zhao, 2012; Zhao et al., 2010). As a result, brown rice and its products, such as brown rice syrup, have a higher concentration of arsenic compared to white rice food products (Gundert‐Remy et al., 2015; Torres‐Escribano et al., 2008). This is of potential concern, because arsenic exposure—even at lower levels that do not cause acute toxicity—has been linked to a number of human health risks, including multiple cancers and cardiovascular disease.

Arsenic, a metalloid with atomic number 33, occurs naturally in the earth's crust and in water. Rice takes up nearly 10 times more soilborne arsenic than other grains: a result of its production method in flooded paddies (Schoof et al., 1999; Williams, Villada, et al., 2007). Arsenic is taken up by rice and other grains via the roots, from whence it translocates throughout the plant. Crucially, it builds up in the consumable part of rice: the grain (Feng Ma et al., 2008; Meharg & Zhao, 2012; Zhao et al., 2010). The rice grain consists of multiple parts that are eaten, leading to the distinction between brown and white rice. Arsenic, specifically inorganic arsenic—the bioavailable form that is most harmful to people, is not evenly distributed throughout the rice plant or the rice grain. Arsenic compounds exist in more than 100 types, and their toxicity depends on their chemical composition and oxidation state (see Table 1).

**TABLE 1:** Arsenic species found in rice.

Name	Abbr.	Chem. form	Toxicity	Grain conc.	Plant conc.
Arsenite	As(III)	Inorganic	High	Bran	Root
Arsenate	As(V)	Inorganic	Medium	Bran	Root
Total inorganic (AsIII + AsV)	iAS	Inorganic	High	Bran	Root
Monomethylarsonic acid	MMA	Organic	Medium	Throughout	Root
Dimethylarsinic acid	DMA	Organic	Medium	Throughout	Shoot
Total arsenic	tAS	Organic and inorganic	Medium	Bran	Root > straw > husk > grain

*Source*: Abedi and Mojiri (2020), Abedin, Cresser, et al. (2002), Bhattacharya et al. (2010), Carey et al. (2010), D'Amato et al. (2004), Islam et al. (2016), Lombi et al. (2009), Meharg, Lombi et al. (2008), Mir et al. (2007), Pasias et al. (2013), Pizarro et al. (2003), Rahman et al. (2007), Reid et al. (2020), Smith et al. (2009), Sun et al. (2008), Syu et al. (2015), Wu et al. (2016), Yim et al. (2017), Zheng et al. (2011).

There are four main species of arsenic found in rice: As(III) and As(V), inorganic forms; and monomethylarsonic acid and dimethylarsinic acid; organic forms. Organic arsenic is generally considered far less toxic than inorganic arsenic (Juhasz et al., 2006; Lombi et al., 2009; Mishra et al., 2023; Murray et al., 2012). Arsenic in rice is concentrated differentially within the plant, generally along the trend of root > straw > husk > bran > endosperm (Abedin, Cresser, et al., 2002; Bhattacharya et al., 2010; Rahman et al., 2007), with higher total and inorganic arsenic content in the outer parts of the rice grain (Lombi et al., 2009; Smith et al., 2009; Sun et al., 2008; Syu et al., 2015; Wu et al., 2016; Zheng et al., 2011).

Globally, it has been established that rice consumption increases arsenic exposure, especially in Asian nations where rice consumption and arsenic concentrations are higher (Banerjee et al., 2013; Meharg et al., 2009; WHO & UNICEF, 2018). Arsenic in rice is an issue that has also received increasing attention in the United States, following work that brought to light the high amount of arsenic found within rice‐based baby foods (Halloran & Rogers, 2018; Hirsch, 2018). The realization of the risk to public health posed by arsenic in food, especially rice‐based products, prompted regulatory action from the US Food and Drug Administration (FDA) (FDA, 2020, 2021). Arsenic content in water is already monitored and regulated to protect the public from potential exposure via drinking water consumption. The new FDA Closer to Zero Action Plan will soon set action levels for arsenic content in food products. Current regulatory measures for arsenic in food and water can be seen in Table 2.

**TABLE 2:** Selected maximum limits of arsenic for consumption in food and water.

Agency (Refs.)	Product	Type	Regulatory limit (ppb)
FDA (2023)	Apple juice	Inorganic arsenic	10
FDA (2020)	Rice cereal for infants	Inorganic arsenic	100
FDA (2005)	Bottled water	Total arsenic	10
EPA (2001)	Drinking water	Total arsenic	10
WHO (2008)	Drinking water	Total arsenic	10
European Union (1998)	Drinking water	Total arsenic	10
JECFA (2001)	Edible fats and oils	Total arsenic	100
JECFA (2001)	Rice, husked	Inorganic arsenic	350
JECFA (2001)	Rice, polished	Inorganic arsenic	200
JECFA (2001)	Salt, food grade	Total arsenic	500

How do concerns regarding arsenic in brown rice weigh against the purported nutritional benefits of the bran and germ in brown rice described above? These factors contribute to the perceived nutritional superiority of brown rice and lead to the promotion of brown rice instead of white rice in nutrition discourse, including by domestic officials through national dietary guidelines that promote whole grains (Mohan et al., 2017). Accordingly, customer education of the benefits of brown rice, especially focused on health and nutrition, may contribute to increasing consumer purchasing and consumption of brown rice in the United States (David et al., 2020; Gyawali et al., 2022; Mohan et al., 2017; Sudha et al., 2013). However, there is very little discussion of higher arsenic content in brown rice compared with white rice. This juxtaposition poses the question of how consumer behavior and public health/dietary discourse may change if this difference between brown and white rice is more fully explored. Perhaps the increased arsenic exposure level of brown rice over white rice is of grave concern, or perhaps the difference is negligible and of little concern.

In this article, we attempt to shed light on this topic by assessing arsenic exposure in the US population from brown rice versus white rice consumption. We first review the evidence for the thesis that brown rice is more healthful than white rice. We then estimate dietary exposure to inorganic arsenic from brown rice compared to white rice. Finally, we discuss the current state of research focusing on arsenic content and rice varieties and suggest avenues for future research.

## 2 METHODS AND DATA

First, we conducted an extensive literature review on the nutritional aspects of brown and white rice. Then we estimate arsenic exposure through brown versus white rice in the United States. We draw from the “What we eat in America” database of the US Environmental Protection Agency (EPA) and Joint Institute of Food Science and Applied Nutrition (JIFSAN) (US Environmental Protection Agency/Joint Institute for Food Safety and Applied Nutrition, 2023) to gain an understanding of how much rice the average American is consuming. The database draws from the dietary intake component of the National Health and Nutrition Examination Survey conducted by the US Department of Agriculture and the US Departments of Health and Human Services. It utilizes 24‐h dietary recall data to assess the diet of a nationally representative sample of children and adults in the United States (EPA/JIFSAN, 2023). Using mean rice intake values, we can examine what the average daily dose (ADD) using the formula of:

ADD=A×IR/BW

where ADD is the average daily dose/intake of arsenic measured in µg As per kg bodyweight per day (µg/kg bw/day), *A* is the arsenic level (total or inorganic) in US rice (either brown or white), calculated in Table 4, IR is the mean daily amount of rice (total, brown, or white) consumed by an age cohort measured in kilograms per day, calculated in Tables 5 and 6, and BW is the average body weight of a given age cohort in the United States in kg. With this calculation, four possible types of values can be produced for analysis: (1) arsenic exposure if all rice consumed was white rice; (2) exposure if all rice was brown rice; (3) exposure when the type of rice consumed was explicitly stated as white; or (4) explicitly stated as brown rice.

## 3 RESULTS

The nutritional benefits of brown rice have been extensively documented in empirical studies (Mir et al., 2020; Saleh et al., 2019; Wu et al., 2013; see Table 3 for more references). Many of the beneficial nutrients of brown rice are lost in milling and processing of white rice, where the bran and germ are wholly or partially removed, leaving the white endosperm behind (Narukawa et al., 2012; Saleh et al., 2019). The nutritional benefits retained by brown rice lead to a public perception of the grain as more healthy than white rice (Mir et al., 2016, 2020). Despite these benefits, it is well documented that brown rice is not nearly as commonly consumed as white rice on a domestic or global scale (Gondal et al., 2021; Saleh et al., 2019; Selvam et al., 2017).

**TABLE 3:** Documented benefits and risks of brown and white rice.

Brown rice	
*Benefits*	*Risks/Drawbacks*
Increased vitamins, minerals, fiber, and other nutrients (Menon et al., 2021; Mir et al., 2020; Narukawa et al., 2012; Rathna et al., 2019; Saleh et al., 2019)	Higher price (Abdul et al., 2012; Hoek et al., 2017; Selvam et al., 2017)
Growing market share (Rathna Priya et al., 2019; Romero, 2011; Selvam et al., 2017; Wu et al., 2018)	Increased arsenic (total and inorganic) content due to rice bran (Abedin, Cotter‐Howells, et al., 2002; Bhattacharya et al., 2010; Lombi et al., 2009; Rahman et al., 2007; Smith et al., 2009; Wu et al., 2016; Zheng et al., 2011)
Socially desirable/perceived as more healthy (Mir et al., 2016, 2020; Mohan et al., 2017; Romero, 2011; Selvam et al., 2017; Su et al., 2023; Wu et al., 2018)	Less desirable (taste/cooking time/storage) among some populations (Gondal et al., 2021; Saleh et al., 2019; Selvam et al., 2017)
Documented health benefits (diminished cancer risk, lower cholesterol, reduced hypertension and obesity, and aiding cardiovascular disease, metabolic disorders, osteoporosis, and diabetes) (Aune et al., 2016; Hudson et al., 2000; Kazemzadeh et al., 2014; Li et al., 2011; Shimabukuro et al., 2014; Yu et al., 2022)	Harmful health outcomes linked to arsenic (genetic damage and increased cancer risk) (Banerjee et al., 2013; Oberoi et al., 2014; Signes‐Pastor et al., 2019; Sofuoglu et al., 2014)
Decreased environmental/labor burden (Javier, 2004; Lee et al., 2019; Mojica & Reforma, 2010; Roy et al., 2011; Saleh et al., 2019; Su et al., 2023)	

White rice	
*Benefits*	*Risks/Drawbacks*
More affordable (Abdul Hadi et al., 2012; Hoek et al., 2017; Selvam et al., 2017)	Decreased vitamins, minerals, fiber, and other nutrients (Menon et al., 2021; Mir et al., 2020; Narukawa et al., 2012; Rathna Priya et al., 2019; Saleh et al., 2019)
Appeals to wider variety of customer/dominant market share (Gondal et al., 2021; Rathna Priya et al., 2019; Romero, 2011; Saleh et al., 2019; Selvam et al., 2017; Wu et al., 2018)	Perceived as less healthy (Mir et al., 2016, 2020; Mohan et al., 2017; Romero, 2011; Selvam et al., 2017; Su et al., 2023; Wu et al., 2018)
Decreased arsenic (total and inorganic) content due to processing (Abedin, Cotter‐Howells, et al., 2002; Bhattacharya et al., 2010; Lombi et al., 2009; Rahman et al., 2007; Smith et al., 2009; Wu et al., 2016; Zheng et al., 2011)	Increased environmental/labor burden (Javier, 2004; Lee et al., 2019; Mojica & Reforma, 2010; Roy et al., 2011; Saleh et al., 2019; Su et al., 2023)
Less documented negative health outcomes due to arsenic consumption (Banerjee et al., 2013; Oberoi et al., 2014; Signes‐Pastor et al., 2019; Sofuoglu et al., 2014)	

### 3.1 Global and domestic As levels in rice

The reduction in arsenic content from postharvest practices (polishing and parboiling) allows for the comparison of documented arsenic levels found in rice grains focusing on the comparison of brown and white rice. In Table 4, we show the average values for studies that explicitly compare the arsenic content of brown and white rice grains, with some studies specifically noting arsenic levels in rice bran.

**TABLE 4:** Mean values of studies documenting arsenic content in white and brown rice.

	White rice			Brown rice			Bran		
Abbr. citation	tAS (µg/kg)	iAS	iAS%	tAS (µg/kg)	iAS	iAS%	tAS (µg/kg)	iAS	iAS%
Atiaga‐Franco et al. (2019)	0.174	–	–	0.232	–	–	–	–	–
Chen et al. (2018)	0.120	0.070	–	0.220	0.110	58	–	–	–
Choi et al. (2014)	0.092	–	–	0.101	–	–	–	–	–
Fransisca et al. (2015)	0.105	–	–	0.135	–	–	–	–	–
Jo and Todorov (2019), ^*^	0.205	–	–	0.262	–	–	0.860	–	–
LaHue et al. (2016), ^*^	0.199	–	–	0.257	–	–	–	–	–
Lombi et al. (2009)	0.540	0.170	32	–	–	–	6.240	3.470	56
Meharg, Lombi, et al. (2008), ^*^	0.280	0.110	39	0.440	0.170	45	–	–	–
Naito et al. (2015)	0.250	0.683	97	0.156	0.128	96	0.157	1.107	113
Narukawa et al. (2012)	0.107	0.092	–	0.173	0.156		0.725	–	–
Narukawa et al. (2014)	0.069	0.059	–	0.239	0.208	–	–	–	–
Nishimura et al. (2010)	0.160	0.123	87	0.248	0.212	90	–	–	–
Pasias et al. (2013)	0.189	0.053	–	0.189	0.053	–	–	–	–
Rahman et al. (2007)	0.400	–	–	0.800	–	–	1.050	–	–
Rahman et al. (2014)	0.262	0.165	58	0.318	0.276	63	–	–	–
Ren et al. (2006)	0.210	–	–	0.260	–	–	0.770	–	–
Ruangwises et al. (2012)	0.128	0.071	56	0.193	0.125	65	0.896	0.635	71
Signes‐Pastor et al. (2016)	0.235	0.101	57	0.351	0.150	54	–	–	–
Sommella et al. (2013)	0.185	0.080	49	–	–	–	–	–	–
Sun et al. (2008)	0.555	0.210	42	0.763	0.367	50	3.303	1.863	58
Syu et al. (2015)	0.600	–	35	–	–	–	3.230		86
Torres‐Escribano et al. (2008)	0.181	0.085	48	0.199	0.144	73	–	–	–
Williams et al. (2005) (full)	0.154	0.050	27	0.194	0.105	53	–	–	–
“”(USA only)^*^	0.276	0.076	42	0.225	0.092	51	–	–	–
Williams, Raab et al. (2007), ^*^	0.261	–	–	0.205	–	–	–	–	–
Yim et al. (2017)	0.105	0.047	–	0.162	0.096	–	–	–	–
Total mean	0.231	0.136	53	0.277	0.163	65	2.148	1.769	77
(±95% CI)	(±0.057)	(±0.075)	(±10)	(±0.075)	(±0.043)	(±11)	(±1.232)	(±1.217)	(±21)
USA mean^*^	0.244	0.093	33	0.278	0.138	48	0.860	–	–
(±95% CI)	(±0.034)	(±0.003)	(±12)	(±0.082)	(±0.064)	(±6)	(–)		

*Note*: ^*^ = The United States focused study. Evidence is mixed regarding the strength of the relationship between iAS and tAS levels (Khan et al., 2010; Ma et al., 2016; Meharg et al., 2009).

The evidence presented in Table 4 supports previous work noting rice bran has a markedly higher (72.2%–98.3%) concentration of inorganic arsenic than the rice endosperm, otherwise referred to as white rice (Lombi et al., 2009; Sun et al., 2008; Syu et al., 2015). The table presents evidence that the increased concentration of inorganic arsenic in the rice bran leads to elevated levels of arsenic in brown rice compared to white rice. Of critical importance, the increase in arsenic content is predominantly inorganic. This trend is further underlined by the high proportion of inorganic arsenic, 77% (±21), among all of the studies that include the bran in comparative analysis.

Table 4 shows a higher concentration of inorganic arsenic in the rest of the world (53% for white rice and 65% for brown rice), compared to rice in the United States (33% for white rice and 48% for brown rice). This is consistent with previous evidence that demonstrates, noting that the reasons are unclear, a higher concentration of inorganic arsenic in rice from non‐US sources (Meharg et al., 2009; Williams, Raab, et al., 2007; Zavala et al., 2008; Zhao et al., 2013). Concentrations vary greatly ranging from 10% to 90% (Khan et al., 2010; Meharg et al., 2009; Syu et al., 2015; Zavala & Duxbury, 2008; Zhao et al., 2010, 2013); with 54% commonly referenced (Suriyagoda et al., 2018). This is contrasted with rice from the United States generally having lower concentrations of 10%–69% (Heitkemper et al., 2001; Williams et al., 2005; Williams, Raab, et al., 2007), with 40% commonly referenced (Meharg et al., 2009). The elevated inorganic arsenic content from international sources may indicate increased risk in consuming non‐domestic rice for the American public.

Table 4 shows that the overall arsenic level difference is minimal between rice from the United States, 0.224 (±0.034) µg/kg white rice and 0.278 (±0.082) µg/kg brown rice, and the global mean, 0.231 (±0.057) µg/kg white rice and 0.277 (±0.075) µg/kg brown rice. These values, and the mean inorganic arsenic content (USA: 0.093 µg/kg white rice and 0.138 µg/kg brown rice; global: 0.136 µg/kg white rice and 0.163 µg/kg brown rice), are in line, although slightly elevated, from the most commonly referenced mean, with previous studies suggestions for average arsenic levels. Total arsenic in rice grains have broadly been posited to be between 0.02 and 0.9 µg/kg (Bhattacharya et al., 2010; Williams, Raab, et al., 2007), with commonly reference global mean of 0.15 µg/kg (Meharg et al., 2009; Meharg & Zhao, 2012). Our calculated mean arsenic level for US rice also falls within the suggested range of 0.1–0.46 µg/kg (Meharg & Rahman, 2003; Williams, Raab, et al., 2007; Zavala & Duxbury, 2008). Inorganic arsenic also is suggested to range between 0.02 and 0.4 µg/kg (Norton et al., 2013; Sun et al., 2008; Torres‐Escribano et al., 2008; Williams, Raab, et al., 2007), with a commonly referenced global mean of 0.1 µg/kg (Meharg et al., 2009; Meharg & Zhao, 2012). It is important to note that the mean calculated values in our analysis are only for studies that explicitly compare brown and white rice. The absolute arsenic values presented are not meant to be a broad declaration of the global and domestic values for all of rice; rather the mean values are used for comparative purposes to estimate how the arsenic levels differ between brown and white rice. The evidence shown in Table 4 is therefore in line with the previous widely held assumption that brown rice is higher in arsenic than white rice and therefore can potentially pose more of a health risk from consumptive exposure. The elevated mean values of arsenic in brown rice compared to white rice can now also be quantified to estimate daily dose exposure to arsenic in the United States.

### 3.2 Average daily dose of As from rice

Table 5 provides data on rice consumption in the United States, by age group and by the total US population versus the group that specifically states eating rice on a regular basis. These data are compiled from the “What We Eat in America” database.

**TABLE 5:** Intake levels of rice for total population and “Eaters only” subpopulation.

	Total population				“Eaters only” subpopulation		
	*n*	Eating %	Mean	95% CI	*n*	Mean	95% CI
Age group			(g/kg bw/day)			(g/kg bw/day)	
0–6 month	1204	30.23	0.52	(0.45, 0.59)	364	1.60	(1.41, 1.79)
6–24 month	2632	72.80	0.81	(0.75, 0.87)	1916	1.09	(1.01, 1.17)
24–60 month	3436	71.30	0.55	(0.50, 0.60)	2450	0.77	(0.71, 0.83)
5–18 years	12,654	68.59	0.25	(0.23, 0.27)	8679	0.36	(0.33, 0.39)
18–60 years	20,006	71.85	0.27	(0.26, 0.28)	14,375	0.36	(0.35, 0.37)
60+ years	9414	70.33	0.14	(0.13, 0.15)	6621	0.20	(0.19, 0.21)
All ages	49,346	69.72	0.27	(0.26, 0.28)	34,405	0.37	(0.35, 0.39)

*Source*: EPA/JIFSAN (US Environmental Protection Agency/Joint Institute for Food Safety and Applied Nutrition) (2023). What we eat in America‐Food Commodity Intake Database 2005–10. https://fcid.foodrisk.org/percentiles.php.

This table demonstrates that children under 5 are, on average, the heaviest consumers per unit bodyweight of rice. The amounts are as follows: for the total population: 0.52 (±0.07), 0.81 (±0.06), 0.55 (±0.05) g/kg bw/day; eaters only: 1.60 (±0.19), 1.09 (±0.08), 0.77 (±0.06) g/kg bw/day; consuming a higher relative amount of rice food products compared to older populations (total population: 0.25 (±0.02), 0.27 (±0.01), 0.14 (±0.01) g/kg bw/day; eaters only: 0.36 (±0.03), 0.36 (±0.01), 0.20 (±0.01) g/kg bw/day). This is notable because the documented populations that are especially vulnerable (due to elevated rice consumption or susceptibility to toxicity) to arsenic exposure from rice include infants and young children, along with Asian immigrant populations, people consuming a gluten‐free diet, and those with diets that have a poor nutritional value (poorer and/or food insecure populations) (Batres‐Marquez & Jensen, 2005; Farzan et al., 2013; Meharg, Sun, et al., 2008; Vahter, 2008). These elevated consumptive levels are additionally concerning because rice consumption statistics often underestimate consumption by the very young, those close to or in poverty, and ethnic groups (like Asians and Native American) with high rates of consumption (Batres‐Marquez & Jensen, 2005). It is also notable that consumption among the oldest age cohort declines (total population: 0.14 (±0.01) g/kg bw/day; eaters only: 0.20 (±0.01) g/kg bw/day) compared to the average rice consumption (total population: 0.27 (±0.01) g/kg bw/day; eaters only: 0.37 (±0.02) g/kg bw/day).

Table 6 shows the total amount of brown rice and white rice consumption by age group and by the total US population versus “eaters only” of rice.

**TABLE 6:** Intake levels of brown and white rice for total population and “Eaters only” subpopulation (infant food included).

	Total population				“Eaters only” subpopulation		
			Mean			Mean	
Age group	*n*	Eating %	(g/kg bw/day)	95% CI	*n*	(g/kg bw/day)	95% CI
**Brown rice**							
0–6 month	1204	0.08	<0.005	(0, <0.005)	1	0.16	(0.16, 0.16)
6–24 month	2632	4.10	0.13	(0.10, 0.16)	108	2.14	(1.75, 2.53)
24–60 month	3436	3.67	0.04	(0.03, 0.05)	126	0.86	(0.67, 1.05)
5–18 years	12,654	2.35	0.02	(0.02, 0.02)	297	0.64	(0.56, 0.72)
18–60 years	20,006	4.57	0.02	(0.02, 0.02)	914	0.32	(0.30, 0.34)
60+ years	9414	4.85	0.01	(0.01, 0.01)	457	0.26	(0.23, 0.29)
All ages	49,346	3.86	0.02	(0.02, 0.02)	1903	0.40	(0.38, 0.42)
**White rice**							
0–6 month	1204	1.33	0.01	(0.01, 0.01)	16	0.71	(0.56, 0.86)
6–24 month	2632	24.24	0.32	(0.29, 0.35)	638	1.51	(1.40, 1.62)
24–60 month	3436	29.74	0.44	(0.39, 0.49)	1022	1.55	(1.44, 1.66)
5–18 years	12,654	23.74	0.20	(0.18, 0.22)	3004	0.94	(0.89, 0.99)
18–60 years	20,006	36.08	0.24	(0.23, 0.25)	7219	0.68	(0.66, 0.70)
60+ years	9414	27.07	0.12	(0.11, 0.13)	2548	0.46	(0.44, 0.48)
All ages	49,346	29.28	0.22	(0.21, 0.23)	14,447	0.72	(0.70, 0.74)

*Source*: EPA/JIFSAN (US Environmental Protection Agency/Joint Institute for Food Safety and Applied Nutrition) (2023). What we eat in America‐ Food Commodity Intake Database 2005–2010. https://fcid.foodrisk.org/percentiles.php.

Table 6 shows higher daily intake of brown rice, 2.14 (±0.39) g/kg bw/day, among children 6–24 months of age. It also shows higher white rice consumption among children, especially ages 6–60 months, 1.51 (±0.11) and 1.55 (±0.11) g/kg bw/day. Both of these trends demonstrate areas of potential heightened exposure to arsenic via rice consumption. Using these mean intake values, we can examine what the ADD of each respective age cohort in µg As per kg bodyweight per day (µg/kg bw/day).

With this calculation four possible types of values can be produced for analysis. The first, shown in Table 7, is the calculation of daily intake of arsenic (total and inorganic) if all of the rice products that were consumed by Americans were derived from white rice. This presents the lower limit of the estimation of daily arsenic exposure from total rice product consumption using the data presented in Table 5. The highest value for inorganic arsenic daily intake in this table is shown to be the youngest age cohort of 0–6 months, 0.149 (±0.018) µg/kg bw/day, demonstrating the heightened vulnerability of infants even under the lowest possible scenario for arsenic exposure.

**TABLE 7:** Total consumption of rice at lowest & highest (white and brown rice) level arsenic exposure.

		Total population					“Eaters only” subpopulation				
			tAS intake—daily		iAS intake—daily			tAS intake—daily		iAS intake—daily	
Age group	Avg. wt^	Mean intake	Mean	95% CI	Mean	95% CI	Mean intake	Mean	95% CI	Mean	95% CI
Unit	Kg	g/kg bw/day	µg/kg		µg/kg		g/kg bw/day	µg/kg		µg/kg	
**Highest (brown rice) level**											
0–6 month	6.219	0.52	0.145	(0.125, 0.164)	0.072	(0.062, 0.081)	1.60	0.445	(0.392, 0.498)	0.221^*^	(0.195, 0.247)
6–24 month	11.528	0.81	0.225	(0.209, 0.242)	0.112	(0.104, 0.120)	1.09	0.303	(0.281, 0.325)	0.150	(0.139, 0.161)
24–60 month	18.554	0.55	0.153	(0.139, 0.167)	0.076	(0.069, 0.083)	0.77	0.214	(0.197, 0.231)	0.106	(0.098, 0.115)
5–18 years	47.606	0.25	0.070	(0.064, 0.075)	0.035	(0.032, 0.037)	0.36	0.100	(0.092, 0.108)	0.050	(0.046, 0.054)
18–60 years	77.895	0.27	0.075	(0.072, 0.078)	0.037	(0.036, 0.039)	0.36	0.100	(0.097, 0.103)	0.050	(0.048, 0.051)
60+ years	75.230	0.14	0.039	(0.036, 0.042)	0.019	(0.018, 0.021)	0.20	0.056	(0.053, 0.058)	0.028	(0.026, 0.029)
All ages	53.924	0.27	0.075	(0.072, 0.078)	0.037	(0.036, 0.039)	0.37	0.103	(0.097, 0.108)	0.051	(0.048, 0.054)
											
**Lowest (white rice) level**											
0–6 month	6.219	0.52	0.127	(0.110, 0.144)	0.048	(0.042, 0.055)	1.60	0.390	(0.344, 0.437)	0.149	(0.131, 0.166)
6–24 month	11.528	0.81	0.198	(0.183, 0.212)	0.075	(0.070, 0.081)	1.09	0.266	(0.246, 0.285)	0.101	(0.094, 0.109)
24–60 month	18.554	0.55	0.134	(0.122, 0.146)	0.051	(0.047, 0.056)	0.77	0.188	(0.173, 0.203)	0.072	(0.066, 0.077)
5–18 years	47.606	0.25	0.061	(0.056, 0.066)	0.023	(0.021, 0.025)	0.36	0.088	(0.081, 0.095)	0.033	(0.031, 0.036)
18–60 years	77.895	0.27	0.066	(0.063, 0.068)	0.025	(0.024, 0.026)	0.36	0.088	(0.085, 0.090)	0.033	(0.033, 0.034)
60+ years	75.230	0.14	0.034	(0.032, 0.037)	0.013	(0.012, 0.014)	0.20	0.049	(0.046, 0.051)	0.019	(0.017, 0.020)
All ages	53.924	0.27	0.066	(0.063, 0.068)	0.025	(0.024, 0.026)	0.37	0.090	(0.085, 0.095)	0.034	(0.033, 0.036)

*Note*: tAS = 0.244; iAS = 0.093 µg/kg. ^*^ = Value exceeds previous recommended safe exposure limit of 0.21 µg/kg bw/day. ^ = Data Source: Fryar et al. (2021). Anthropometric reference data for children and adults: the United States, 2015–2018. https://www.cdc.gov/nchs/data/series/sr_03/sr03‐046‐508.pdf.

The exposure assessment in Table 7 shows how much arsenic (total and inorganic) if all rice products (rice grains, rice cereal, rice syrup, etc.) consumed by Americans were derived from brown rice. This presents the upper limit of the estimation of daily arsenic exposure from the consumption data displayed in Table 5. In Table 8, the value that is the most concerning is a value of 0.221 (±0.026) µg/kg bw/day for children in the 0–6 months old age group. This value exceeds the previous recommended safe daily dose for inorganic arsenic of 0.21 µg/kg bw/day proposed by JECFA (2011). Broadly: because of higher rice intake per unit bodyweight, children under 5 years of age have a substantially greater intake than the overall mean daily exposure for the general population, 0.051 (±0.003) µg/kg bw/day.

**TABLE 8:** Arsenic exposure for daily consumption of brown and/or white rice.

		Total population					“Eaters only” subpopulation				
			tAS intake—daily		iAS intake—daily			tAS intake—daily		iAS intake—daily	
Age group	Avg. wt^	Mean intake	Mean	95% CI	Mean	95% CI	Mean intake	Mean	95% CI	Mean	95% CI
Unit	kg	g/kg bw/day	µg/kg		µg/kg		g/kg bw/day	µg/kg		µg/kg	
**Brown rice**											
0–6 month	6.219	<0.005	<0.005	(0, <0.005)	<0.005	(0, <0.005)	0.16	0.044	(0.044, 0.044)	0.022	(0.022, 0.022)
6–24 month	11.528	0.13	0.036	(0.028, 0.044)	0.018	(0.014, 0.022)	2.14	0.595	(0.487, 0.703)	0.295^*^	(0.242, 0.349)
24–60 month	18.554	0.04	0.011	(0.008, 0.014)	0.006	(0.004, 0.007)	0.86	0.239	(0.186, 0.292)	0.119	(0.092, 0.145)
5–18 years	47.606	0.02	0.006	(0.006, 0.006)	0.003	(0.003, 0.003)	0.64	0.178	(0.156, 0.200)	0.088	(0.077, 0.099)
18–60 years	77.895	0.02	0.006	(0.006, 0.006)	0.003	(0.003, 0.003)	0.32	0.089	(0.083, 0.095)	0.044	(0.041, 0.047)
60+ years	75.230	0.01	0.003	(0.003, 0.003)	0.001	(0.001, 0.001)	0.26	0.072	(0.064, 0.081)	0.036	(0.032, 0.040)
All ages	53.924	0.02	0.006	(0.006, 0.006)	0.003	(0.003, 0.003)	0.40	0.111	(0.106, 0.117)	0.055	(0.052, 0.058)
**White rice**											
0–6 month	6.219	0.01	0.002	(0.002, 0.002)	0.001	(0.001, 0.001)	0.71	0.173	(0.137, 0.210)	0.066	(0.052, 0.080)
6–24 month	11.528	0.32	0.078	(0.071, 0.085)	0.030	(0.027, 0.033)	1.51	0.368	(0.342, 0.395)	0.140	(0.130, 0.151)
24–60 month	18.554	0.44	0.107	(0.095, 0.120)	0.041	(0.036, 0.046)	1.55	0.378	(0.368, 0.405)	0.144	(0.140, 0.154)
5–18 years	47.606	0.20	0.049	(0.044, 0.054)	0.019	(0.017, 0.020)	0.94	0.229	(0.217, 0.242)	0.087	(0.083, 0.092)
18–60 years	77.895	0.24	0.059	(0.056, 0.061)	0.022	(0.021, 0.023)	0.68	0.166	(0.161, 0.171)	0.063	(0.061, 0.065)
60+ years	75.230	0.12	0.029	(0.027, 0.032)	0.011	(0.010, 0.012)	0.46	0.112	(0.107, 0.117)	0.043	(0.041, 0.045)
All ages	53.924	0.22	0.054	(0.051, 0.056)	0.020	(0.020, 0.021)	0.72	0.176	(0.171, 0.181)	0.067	(0.065, 0.069)

*Note*: Brown rice tAS = 0.278 and iAS = 0.138 µg/kg. White rice tAS = 0.244 and iAS = 0.093 µg/kg. ^*^ = Value exceeds previous recommended safe exposure limit of 0.21 g/kg bw/day. ^ = Data Source: Fryar et al. (2021). Anthropometric reference data for children and adults: the United States, 2015–2018. https://www.cdc.gov/nchs/data/series/sr_03/sr03‐046‐508.pdf.

Table 8 shows imputed arsenic exposure by age group for brown rice versus white rice eaters in the United States. This evidence presents a specific narrative for brown and white rice that differs depending on the respective age cohort. The daily intake of inorganic arsenic exceeds 0.295 (±0.64) µg/kg bw/day, previously considered safe levels (0.21 µg/kg bw/day) for the brown rice “eaters‐only” subpopulation for children aged 6–24 months. Elevated intake of inorganic arsenic is also observed among children in the “eaters‐only” subpopulation for white and brown rice for all children (<18 years old), but especially among children ages 6–60 months. Children aged 6 months and under demonstrate lower rates of consumption and exposure, likely because they are not eating solid foods and the solid foods they may consume are rarely defined as a distinct white or brown rice product. It is important to point out that, for adult subpopulations, the inorganic arsenic exposure from specific brown and white products is relatively low, ranging from 0.036 to 0.063 µg/kg bw/day, especially among older (60+) adults.

## 4 DISCUSSION

This study sought to shed light on the question of arsenic exposure through brown rice versus white rice, to provide quantitative information assessing the benefit–risk tradeoff of brown rice's purported nutritional benefits versus potentially higher arsenic content. All values for the estimated daily intake of inorganic arsenic fall within the or near the previous assessment by JECFA (2011) that demonstrated intakes of 0.08–1.19 µg/kg bw/day for children and adults in the United States. The values reported in the analysis indicate that there is a potential risk to harmful exposure to arsenic from brown rice among children under the age of 5. However, the daily inorganic arsenic exposure for most Americans within the analysis did not rise to a level that was a concern to pose elevated risks of harmful health outcomes. This finding was in agreement with previous evidence that finds limited concentrations and population‐level exposure risks associated with arsenic and US rice‐based foods (Consumer Reports, 2012; FDA, 2013; JECFA, 2012).

This exposure assessment is only one side of the equation when examining the potential trade‐offs between brown and white rice consumption. Even if arsenic levels are slightly higher in brown rice than white rice, more research is needed to demonstrate if the potential risks from this exposure are mitigated in part by the potential nutritional benefits provided by the rice bran. It is known, for example, that certain micronutrients may biotransform inorganic arsenic to an organic form that is more readily excreted.

Over a lifetime, chronic arsenic exposure may increase the risk of multiple types of cancer, mainly skin, lung, and bladder to levels that present a threat to public health (FDA, 2016; Oberoi et al, 2014; Tsuji et al., 2007). Previous research has shown that health messaging is effective in influencing consumer purchasing patterns for rice (David et al., 2020; Gyawali et al., 2022; Mohan et al., 2017; Sudha et al., 2013). Because of this, it is important to include the potential risks of arsenic exposure in the discussion of health impacts of brown or white rice consumption. The concentrated amount of inorganic arsenic found within the rice bran in analysis challenges a common narrative of rice bran as a health product “super food” because of its increased nutrients and high fiber (Su et al., 2023; Sun et al., 2008). Our analysis shows that not only is this focus on nutritional content limited, but it also agrees with other studies that find elevated risks of arsenic exposure to children that potentially arise from the promotion of these more “health foods” (Meharg, Sun, et al., 2008; Sun et al., 2008).

To add complexity, the increased nutrients, such as fiber, in brown rice also have a mixed relationship with arsenic exposure. Some scholars note that the fibrous components of rice may increase arsenic exposure, with fiber rich diets resulting in greater arsenic bioaccessibility following consumption (Alava et al., 2012; Van de Wiele et al., 2015). Others suggest that the nutrients provided by brown rice may be important to those that chronically exposed to arsenic (Heck et al., 2009; McCarty et al., 2011).

A potential limitation of this study is the variance in the outcomes when measuring arsenic content and proportions in grains utilizing different methods (Meharg, Lombi, et al., 2008; Mir et al., 2007). Another limitation is the necessity of the study to include sources that explicitly reference arsenic concentrations in brown rice or rice bran in comparison with white rice or the rice endosperm. This means that the estimated levels of arsenic concentrations were drawn from a limited sample of sources, introducing the possibility that our estimates are artificially higher or lower than the actual mean values for the rice produced domestically and globally.

The amount of arsenic that ultimately is taken up by a rice grain is highly context specific and depends on the rice genotype, growing conditions (climate, soil, rice cultivar, and potential crop rotation strategy), and crop management practices (water management, input usage, field tillage, and potential intercropping strategies) (Abedin, Cotter‐Howells, et al., 2002; Hua et al., 2011; Islam et al., 2016; Kumarathilaka et al., 2018). Arsenic content in rice varies tremendously depending on geographic location and scale of analysis (up to 6–7 times within countries and 40 times between nations) (Majumder & Banik, 2019; Meharg et al., 2009; Syu et al., 2015).

Even after harvesting rice there are additional factors impacting the arsenic levels in grains, including processing (especially parboiling), milling/polishing, storage, fortification, and cooking/utilization practices (Carey et al., 2015; Mishra et al., 2023; Naito et al., 2015; Panthri & Gupta, 2022; Rahman et al., 2006). Processing location also impacts arsenic content with evidence showing that postharvest strategies do not universally reduce arsenic levels in rice. Cooking, parboiling, and irrigating rice with arsenic contaminated water (often more common in Southeast Asia) will increase the arsenic content found in rice grain (Ackerman et al., 2005; Rahman et al., 2006; Rahman & Hasegawa, 2011). Nevertheless, there is extensive, although not unanimous (Moore et al., 2012), evidence that polishing (removing the pericarp, aleurone layer, and germ making up the rice bran) is effective in substantially reducing the total and inorganic arsenic content of rice (Chowdhury et al., 2019; Mishra et al., 2023; Pedron et al., 2019). For these reasons, it is difficult to compare arsenic levels in rice grains across studies and in order to directly compare arsenic levels in grain. More research is needed to directly compare arsenic levels in rice grains.

Three priority areas for future work include the following:

- Considering nutritional differences, potential food safety concerns, and the impacts on vulnerable populations, an empirical analysis is needed of the cost and benefits to societal public health of consuming brown rice compared to white rice.
- The theoretical and ethical considerations that accompany the discourse of brown or white rice promotion as points of comparison. Perhaps this is a new wave of critical “nutritionism” that goes beyond the initial wave of assumed food content and looks more deeply at unintended food safety and public health effects of food.
- A conceptual policy‐impact analysis of the considerations that rice farmers must take in response to the “Closer to Zero” action plan by the FDA.

## 5 CONCLUSION

Most of the previous scientific and public attention examining arsenic consumption threats to public health has focused on drinking water and not on food (Chappell et al., 1997). However, there is a growing acknowledgement of the importance of examining arsenic exposure via rice consumption as a public health issue in need of an evidence‐based risk analysis approach (Majumder & Banik, 2019; Meharg, 2004; Sauvé, 2014; Schmidt, 2015; Sofuoglu et al., 2014; Su et al., 2023). Our analysis has focused on the aspects of arsenic exposure in brown and white rice that potentially may pose a threat to public food safety. We showed that rice bran is the major contributor of inorganic arsenic in the elevated levels of arsenic observed in brown rice compared to white rice. We showed how US‐based rice and white rice have lower total values and concentrations (percent of total arsenic that is inorganic) of inorganic arsenic compared to brown rice and the global rice supply. Overall, the levels of arsenic in the daily intake from rice for the average adult Americans was of limited concern. We showed how comparative trends demonstrate heightened vulnerability to arsenic (total and inorganic) exposure from rice consumption by American children, especially those under the age of 5. We found the high amount of arsenic exposure risk from children under the age of 5 consuming brown rice and a general risk of exposure, even at the lowest levels of any rice consumption, for children under the age of 6 months. We concluded with a discussion of the need for more research into the potential trade‐offs and relative arsenic exposure risks associated with brown versus white rice, emphasizing food safety concerns balanced with nutritional benefits.

## CONFLICT OF INTEREST STATEMENT

The authors have no conflicts of interest to report.

## Data Availability

Data sharing is not applicable to this article as no new data were created or analyzed in this study.

## References

[risa70008-bib-0001] Abdul Hadi, A. H. , Selamat, J. , Shamsudin, M. N. , Radam, A. , & Ab Karim, M. (2012). Consumers’ demand and willingness to pay for rice attributes in Malaysia. International Food Research Journal, 19, 363–369.

[risa70008-bib-0002] Abedi, T. , & Mojiri, A. (2020). Arsenic uptake and accumulation mechanisms in rice species. Plants, 9(2), 1–17. 10.3390/plants9020129 PMC7076356 31972985

[risa70008-bib-0003] Abedin, J. , Cresser, M. S. , Meharg, A. A. , Feldmann, J. , & Cotter‐Howells, J. (2002). Arsenic accumulation and metabolism in rice (*Oryza sativa* L.). Environmental Science and Technology, 36(5), 962–968. 10.1021/es0101678 11918027

[risa70008-bib-0004] Abedin, Md. J. , Cotter‐Howells, J. , & Meharg, A. A. (2002). Arsenic uptake and accumulation in rice (*Oryza sativa* L.) irrigated with contaminated water. Plant and Soil, 240(2), 311–319. 10.1023/A:1015792723288

[risa70008-bib-0005] Ackerman, A. H. , Creed, P. A. , Parks, A. N. , Fricke, M. W. , Schwegel, C. A. , Cred, J. T. , Heitkemper, D. T. , & Vela, N. P. (2005). Comparison of a chemical enzymatic extraction of arsenic from rice and an assessment of the arsenic absorption rom contaminated water by cooked rice. Environmental Science and Technology, 39, 5241–5246. 16082952 10.1021/es048150n

[risa70008-bib-0006] Alava, P. , Du Laing, G. , Tack, F. , & Van De Wiele, T. (2012). Effect of diet on bio accessibility and biotransformation of arsenic. In Understanding *the geological and medical interface of arsenic*‐As 2012 (pp. 306–308). CRC Press.

[risa70008-bib-0007] Atiaga‐Franco, L. O. , Otero, L. X. , Gallego‐Pico, A. , Escobar‐Castaneda, A. L. , Bravo‐Yague, C. J. , & Carrera‐Villacres, D. (2019). Analysis of total arsenic content in purchased rice from Ecuador. Czech Journal of Food Sciences, 37(6), 425–431. https://cjfs.agriculturejournals.cz/artkey/cjf‐201906‐0005.php

[risa70008-bib-0008] Aune, D. , Keum, N. , Giovannucci, E. , Fadnes, L. T. , Boffetta, P. , Greenwood, D. C. , Tonstad, S. , Vatten, L. J. , Riboli, E. , & Norat, T. (2016). Whole grain consumption and risk of cardiovascular disease, cancer, and all cause and cause specific mortality: Systematic review and dose‐response meta‐analysis of prospective studies. BMJ, 353, i2716. 10.1136/bmj.i2716 27301975 PMC4908315

[risa70008-bib-0009] Banerjee, M. , Banerjee, N. , Bhattacharjee, P. , Mondal, D. , Lythgoe, P. R. , Martínez, M. , Pan, J. , Polya, D. A. , & Giri, A. K. (2013). High arsenic in rice is associated with elevated genotoxic effects in humans. Scientific Reports, 3(2195), 1–8. 10.1038/srep02195 PMC6505394 23873074

[risa70008-bib-0010] Batres‐Marquez, S. , & Jensen, H. (2005). Rice Consumption in the United States: New evidence from food consumption surveys. Iowa State University. 10.1016/j.jada.2009.07.010 19782171

[risa70008-bib-0011] Batres‐Marquez, S. P. , Jensen, H. H. , & Upton, J. (2009). Rice consumption in the United States: Recent evidence from food consumption surveys. Journal of the American Dietetic Association, 109(10), 1719–1727. 10.1016/j.jada.2009.07.010 19782171

[risa70008-bib-0012] Bhattacharya, P. , Samal, A. C. , Majumdar, J. , & Santra, S. C. (2010). Accumulation of arsenic and its distribution in rice plant (*Oryza sativa* L.) in Gangetic West Bengal, India. Paddy and Water Environment, 8(1), 63–70. 10.1007/s10333-009-0180-z

[risa70008-bib-0013] Carey, A. M. , Scheckel, K. G. , Lombi, E. , Newville, M. , Choi, Y. , Norton, G. J. , Charnock, J. M. , Feldmann, J. , Price, A. H. , & Meharg, A. A. (2010). Grain unloading of arsenic species in rice. Plant Physiology, 152(1), 309–319. 10.1104/pp.109.146126 19880610 PMC2799365

[risa70008-bib-0014] Carey, M. , Jiujin, X. , Gomes Farias, J. , & Meharg, A. A. (2015). Rethinking rice preparation for highly efficient removal of inorganic arsenic using percolating cooking water. PLoS ONE, 10(7), e0131608. 10.1371/journal.pone.0131608 26200355 PMC4511802

[risa70008-bib-0015] Chappell, W. R. , Beck, I. B. D. , Brown, K. G. , Chaney, R. , Cothern, C. R. , Irgolic, K. J. , North, D. W. , Thornton, L. , & Tsongas, T. A. (1997). Inorganic arsenic: A need and an opportunity to improve risk assessment. Environmental Health Perspectives, 105(10), 1060–1067. 9349827 10.1289/ehp.971051060 PMC1470381

[risa70008-bib-0016] Chen, H. , Tang, Z. , Wang, P. , & Zhao, F.‐J. (2018). Geographical variations of cadmium and arsenic concentrations and arsenic speciation in Chinese rice. Environmental Pollution, 238, 482–490. 10.1016/j.envpol.2018.03.048 29602104

[risa70008-bib-0017] Choi, S. H. , Kim, J. S. , Lee, J. Y. , Jeon, J. S. , Kim, J. W. , Russo, R. E. , Gonzalez, J. , Yoo, J. H. , Kim, K. S. , Yang, J. S. , & Park, K. S. (2014). Analysis of arsenic in rice grains using ICP‐MS and fs LA‐ICP‐MS. Journal of Analytical Atomic Spectrometry, 29(7), 1233–1237. 10.1039/C4JA00069B

[risa70008-bib-0018] Chowdhury, N. R. , Joardar, M. , Ghosh, S. , Bhowmick, S. , & Roychowdhury, T. (2019). Variation of arsenic accumulation in paddy plant with special reference to rice grain and its additional entry during post‐harvesting technology. In S. Ray (Ed.), Ground water development—Issues and sustainable solutions (pp. 289–303). Springer Singapore. 10.1007/978-981-13-1771-2_17

[risa70008-bib-0019] Consumer Reports . (2012). Results of our tests of rice and rice products. Consumer Reports. https://www.consumerreports.org/cro/magazine/2012/11/arsenic‐in‐your‐food/index.htm

[risa70008-bib-0020] D'Amato, M. , Forte, G. , & Caroli, S. (2004). Identification and quantification of major specier of arsenic in rice. Journal of AOAC International, 87, 238–243. 15084106

[risa70008-bib-0021] David, W. , Ardiansyah Budijanto, S. , & Strassner, C. (2020). Sensory evaluation and nutritional information on organic brown rice. Organic Agriculture, 10(2), 243–252. 10.1007/s13165-019-00269-z

[risa70008-bib-0116] EPA . (2001). EPA announces arsenic standard for drinking water of 10 parts per billion. US Department of Health and Human Services.

[risa70008-bib-0022] European Union . (1998). On the quality of water intended for human consumption, Pub. L. No. 98/83/EC, COUNCIL DIRECTIVE 98/83/EC. European Union.

[risa70008-bib-0023] Farzan, S. F. , Karagas, M. R. , & Chen, Y. (2013). In utero and early life arsenic exposure in relation to long‐term health and disease. Toxicology and Applied Pharmacology, 272(2), 384–390. 10.1016/j.taap.2013.06.030 23859881 PMC3783578

[risa70008-bib-0024] FDA . (2013). Analytical *results from inorganic arsenic in rice and rice products sampling* , September 2013. US Food and Drug Administration. http://www.fda.gov/downloads/Food/FoodborneIllnessContaminants/Metals/UCM352467.pdf

[risa70008-bib-0025] FDA . (2016). Risk *assessment report*: Arsen*ic in rice and rice products* . US Food and Drug Administration.

[risa70008-bib-0026] FDA . (2020). US Food and Drug Administration *supporting document for action level for inorganic arsenic in rice cereals for infants* . US Food and Drug Administration.

[risa70008-bib-0027] FDA . (2021). Closer to zero: Reducing childhood exposure to contaminants from foods. US Food and Drug Administration. https://www.fda.gov/food/environmental‐contaminants‐food/closer‐zero‐reducing‐childhood‐exposure‐contaminants‐foods

[risa70008-bib-0114] FDA . (2020). Inorganic *arsenic in rice cereals for infants*: A*ction level guidance for industry* . US Department of Health and Human Services.

[risa70008-bib-0115] FDA . (2023). Action level for inorganic arsenic in apple juice: Guidance for industry. US Department of Health and Human Services.

[risa70008-bib-0136] FDA . (2005). Beverages: Bottled Water, Pub. L. No. 21 CFR Part 165, Federal Register 33694. 15945150

[risa70008-bib-0028] Feng Ma, J. , Yamaji, N. , Mitani, N. , Xu, X.‐Y. , Su , Y.‐H. , Mcgrath, S. P. , & Zhao, F.‐J. (2008). Transporters of arsenite in rice and their role in arsenic accumulation in rice grain. PNAS, 105, 9931–9935. 18626020 10.1073/pnas.0802361105 PMC2481375

[risa70008-bib-0029] Fransisca, Y. , Small, D. M. , Morrison, P. D. , Spencer, M. J. S. , Ball, A. S. , & Jones, O. A. H. (2015). Assessment of arsenic in Australian grown and imported rice varieties on sale in Australia and potential links with irrigation practises and soil geochemistry. Chemosphere, 138, 1008–1013. 10.1016/j.chemosphere.2014.12.048 25577696

[risa70008-bib-0030] Fryar, C. , Carroll, M. , Gu, Q. , Afful, J. , & Ogden, C. (2021). Anthropometric *reference data for children and adults*: United States, 2015–2018: Analyti*cal and epidemiological studies* . US Department of Health and Human Services. https://www.cdc.gov/nchs/data/series/sr_03/sr03‐046‐508.pdf 33541517

[risa70008-bib-0031] Fukagawa, N. K. , & Ziska, L. H. (2019). Rice: Importance for global nutrition. Journal Nutrition Science Vitaminol, 65, S2–S3. http://www.riceassociation.org.uk/content/1/18/types‐ 10.3177/jnsv.65.S2 31619630

[risa70008-bib-0032] Gondal, T. A. , Keast, R. S. J. , Shellie, R. A. , Jadhav, S. R. , Gamlath, S. , Mohebbi, M. , & Liem, D. G. (2021). Consumer acceptance of brown and white rice varieties. Foods, 10(8), 195. 10.3390/foods10081950 34441728 PMC8391279

[risa70008-bib-0033] Gundert‐Remy, U. , Damm, G. , Foth, H. , Freyberger, A. , Gebel, T. , Golka, K. , Röhl, C. , Schupp, T. , Wollin, K. M. , & Hengstler, J. G. (2015). High exposure to inorganic arsenic by food: The need for risk reduction. Archives of Toxicology, 89(12), 2219–2227. 10.1007/s00204-015-1627-1 26586021

[risa70008-bib-0034] Gyawali, P. , Tamrakar, D. , Shrestha, A. , Shrestha, H. , Karmacharya, S. , Bhattarai, S. , Bhandari, N. , Malik, V. , Mattei, J. , Spiegelman, D. , & Shrestha, A. (2022). Consumer acceptance and preference for brown rice—A mixed‐method qualitative study from Nepal. Food Science and Nutrition, 10(6), 1864–1874. 10.1002/fsn3.2803 35702294 PMC9179153

[risa70008-bib-0035] Halloran, J. , & Rogers, J. E. (2018). Consumer reports letter to FDA on heavy metals in baby and toddler food 8‐16‐18. Consumer Reports.

[risa70008-bib-0036] Heck, J. E. , Nieves, J. W. , Chen, Y. , Parvez, F. , Brandt‐Rauf Paul, W. , Graziano , J. H. , Slavkovich, V. , Howe, G. R. , & Ahsan, H. (2009). Dietary intake of methionine, cysteine, and protein and urinary arsenic excretion in Bangladesh. Environmental Health Perspectives, 117(1), 99–104. 10.1289/ehp.11589 19165394 PMC2627873

[risa70008-bib-0037] Heitkemper, D. T. , Vela, N. P. , Stewart, K. R. , & Westphal, C. S. (2001). Determination of total and speciated arsenic in rice by ion chromatography and inductively coupled plasma mass spectrometry. Journal of Analytical Atomic Spectrometry, 16(4), 299–306. 10.1039/b007241i

[risa70008-bib-0038] Hirsch, J. (2018). Heavy metals in baby food: What you need to know . Consumer Reports.

[risa70008-bib-0039] Hoek, A. C. , Pearson, D. , James, S. W. , Lawrence, M. A. , & Friel, S. (2017). Healthy and environmentally sustainable food choices: Consumer responses to point‐of‐purchase actions. Food Quality and Preference, 58, 94–106. 10.1016/j.foodqual.2016.12.008

[risa70008-bib-0040] Hua, B. , Yan, W. , Wang, J. , Deng, B. , & Yang, J. (2011). Arsenic accumulation in rice grains: Effects of cultivars and water management practices. Environmental Engineering Science, 28(8), 591–596. 10.1089/ees.2010.0481

[risa70008-bib-0041] Hudson, E. A. , Dinh, P. A. , Kokubun, T. , Simmonds, M. S. J. , & Gescher, A. (2000). Characterization of potentially chemopreventive phenols in extracts of brown rice that inhibit the growth of human breast and colon cancer cells. Cancer Epidemiology & Prevention, 9, 1163–1170. 11097223

[risa70008-bib-0042] Islam, S. , Rahman, M. M. , Islam, M. R. , & Naidu, R. (2016). Arsenic accumulation in rice: Consequences of rice genotypes and management practices to reduce human health risk. Environment International, 96, 139–155. 10.1016/j.envint.2016.09.006 27649473

[risa70008-bib-0043] Javier, E. Q. (2004, January 18). Let's promote brown rice to combat hidden hunger. Rice Today, 3(1), 38.

[risa70008-bib-0044] JECFA . (2001). Code of practice concerning source directed measures to reduce contamination of foods with chemicals (CXC 49–2001). JECFA.

[risa70008-bib-0045] JECFA . (2011). Arsenic (addendum). In WHO *food additives series* (Vol., 63). WHO.

[risa70008-bib-0046] JECFA . (2012). Report of the *sixth session of the codex committee on contaminants in foods* . Joint FAO/WHO Food Standards Programme Codex Commission.

[risa70008-bib-0047] Jo, G. , & Todorov, T. I. (2019). Distribution of nutrient and toxic elements in brown and polished rice. Food Chemistry, 289, 299–307. 10.1016/j.foodchem.2019.03.040 30955616

[risa70008-bib-0048] Juhasz, A. L. , Smith, E. , Weber, J. , Rees, M. , Rofe, A. , Kuchel, T. , Sansom, L. , & Naidu, R. (2006). In vivo assessment of arsenic bioavailability in rice and its significance for human health risk assessment. Environmental Health Perspectives, 114(12), 1826–1831. 10.1289/ehp.9322 17185270 PMC1764129

[risa70008-bib-0049] Kazemzadeh, M. , Morteza Safavi, S. , Nematollahi, S. , & Nourieh, Z. (2014). Effect of brown rice consumption on inflammatory marker and cardiovascular risk factors among overweight and obese non‐menopausal female adults. International Journal of Preventive Medicine, 5(4), 478–488. 24829736 PMC4018597

[risa70008-bib-0050] Khan, M. A. , Stroud, J. L. , Zhu, Y.‐G. , McGrath, S. P. , & Zhao, F.‐J. (2010). Arsenic bioavailability to rice is elevated in Bangladeshi paddy soils. Environmental Science & Technology, 44(22), 8515–8521. 10.1021/es101952f 20977268

[risa70008-bib-0051] Kumar, S. , Mohanraj, R. , Sudha, V. , Wedick, N. M. , Malik, V. , Hu, F. B. , Spiegelman, D. , & Mohan, V. (2011). Perceptions about varieties of brown rice: A qualitative study from Southern India. Journal of the American Dietetic Association, 111(10), 1517–1522. 10.1016/j.jada.2011.07.002 21963018

[risa70008-bib-0052] Kumarathilaka, P. , Seneweera, S. , Meharg, A. , & Bundschuh, J. (2018). Arsenic accumulation in rice (*Oryza sativa* L.) is influenced by environment and genetic factors. Science of The Total Environment, 642, 485–496. 10.1016/j.scitotenv.2018.06.030 29908507

[risa70008-bib-0053] LaHue, G. T. , Chaney, R. L. , Adviento‐Borbe, M. A. , & Linquist, B. A. (2016). Alternate wetting and drying in high yielding direct‐seeded rice systems accomplishes multiple environmental and agronomic objectives. Agriculture, Ecosystems and Environment, 229, 30–39. 10.1016/j.agee.2016.05.020

[risa70008-bib-0054] Lee, J.‐S. , Sreenivasulu, N. , Hamilton, R. S. , & Kohli, A. (2019). Brown rice, a diet rich in health promoting properties. Journal of Nutritional Science and Vitaminology, 65, S26–S38. 31619639 10.3177/jnsv.65.S26

[risa70008-bib-0055] Li, S.‐C. , Chou, T.‐C. , & Shih, C.‐K. (2011). Effects of brown rice, rice bran, and polished rice on colon carcinogenesis in rats. Food Research International, 44(1), 209–216. 10.1016/j.foodres.2010.10.034

[risa70008-bib-0056] Lombi, E. , Scheckel, K. G. , Pallon, J. , Carey, A. M. , Zhu, Y. G. , & Meharg, A. A. (2009). Speciation and distribution of arsenic and localization of nutrients in rice grains. New Phytologist, 184(1), 193–201. 10.1111/j.1469-8137.2009.02912.x 19549132

[risa70008-bib-0057] Ma, L. , Wang, L. , Jia, Y. , & Yang, Z. (2016). Arsenic speciation in locally grown rice grains from Hunan Province, China: Spatial distribution and potential health risk. Science of the Total Environment, 557–558, 438–444. 10.1016/j.scitotenv.2016.03.051 27016689

[risa70008-bib-0058] Majumder, S. , & Banik, P. (2019). Geographical variation of arsenic distribution in paddy soil, rice and rice‐based products: A meta‐analytic approach and implications to human health. Journal of Environmental Management, 233, 184–199. 10.1016/j.jenvman.2018.12.034 30580115

[risa70008-bib-0059] McCarty, K. M. , Hanh, H. T. , & Kim, K.‐W. (2011). Arsenic geochemistry and human health in South East Asia. Reviews on Environmental Health, 26(1), 71–78. 10.1515/reveh.2011.010 21714384 PMC3128386

[risa70008-bib-0060] Meharg, A. A. (2004). Arsenic in rice—Understanding a new disaster for South‐East Asia. Trends in Plant Science, 9(9), 415–417. 10.1016/j.tplants.2004.07.002 15337490

[risa70008-bib-0061] Meharg, A. A. , Lombi, E. , Williams, P. N. , Scheckel, K. G. , Feldmann, J. , Raab, A. , Zhu, Y. , & Islam, R. (2008). Speciation and localization of arsenic in white and brown rice grains. Environmental Science and Technology, 42(4), 1051–1057. 10.1021/es702212p 18351071

[risa70008-bib-0062] Meharg, A. A. , & Rahman, Md. M. (2003). Arsenic contamination of Bangladesh paddy field soils: Implications for rice contribution to arsenic consumption. Environmental Science & Technology, 37(2), 229–234. 10.1021/es0259842 12564892

[risa70008-bib-0063] Meharg, A. A. , Sun, G. , Williams, P. N. , Adomako, E. , Deacon, C. , Zhu, Y.‐G. , Feldmann, J. , & Raab, A. (2008). Inorganic arsenic levels in baby rice are of concern. Environmental Pollution, 152(3), 746–749. 10.1016/j.envpol.2008.01.043 18339463

[risa70008-bib-0064] Meharg, A. A. , Williams, P. N. , Adomako, E. , Lawgali, Y. Y. , Deacon, C. , Villada, A. , Cambell, R. C. J. , Sun, G. , Zhu, Y. G. , Feldmann, J. , Raab, A. , Zhao, F. J. , Islam, R. , Hossain, S. , & Yanai, J. (2009). Geographical variation in total and inorganic arsenic content of polished (white) rice. Environmental Science and Technology, 43(5), 1612–1617. 10.1021/es802612a 19350943

[risa70008-bib-0065] Meharg, A. , & Zhao, F. (2012). Arsenic & rice. Springer.

[risa70008-bib-0066] Menon, M. , Dong, W. , Chen, X. , Hufton, J. , & Rhodes, E. J. (2021). Improved rice cooking approach to maximise arsenic removal while preserving nutrient elements. Science of the Total Environment, 755, 143341. 10.1016/j.scitotenv.2020.143341 33153748

[risa70008-bib-0067] Mir, K. A. , Rutter, A. , Koch, I. , Smith, P. , Reimer, K. J. , & Poland, J. S. (2007). Extraction and speciation of arsenic in plants grown on arsenic contaminated soils. Talanta, 72(4), 1507–1518. 10.1016/j.talanta.2007.01.068 19071791

[risa70008-bib-0068] Mir, S. A. , Bosco, S. J. D. , Shah, M. A. , & Mir, M. M. (2016). Effect of puffing on physical and antioxidant properties of brown rice. Food Chemistry, 191, 139–146. 10.1016/j.foodchem.2014.11.025 26258713

[risa70008-bib-0069] Mir, S. A. , Shah, M. A. , Bosco, S. J. D. , Sunooj, K. V. , & Farooq, S. (2020). A review on nutritional properties, shelf life, health aspects, and consumption of brown rice in comparison with white rice. In Cereal chemistry (Vol. 97, pp. 895–903). Wiley‐Blackwell. 10.1002/cche.10322

[risa70008-bib-0070] Mishra, S. , Dwivedi, S. , Gupta, A. , & Tiwari, R. K. (2023). Evaluating the efficacy and feasibility of post harvest methods for arsenic removal from rice grain and reduction of arsenic induced cancer risk from rice‐based diet. Science of the Total Environment, 874, 162443. 10.1016/j.scitotenv.2023.162443 36858216

[risa70008-bib-0071] Mohan, V. , Ruchi, V. , Gayathri, R. , Ramya Bai, M. , Shobana, S. , Anjana, R. M. , Unnikrishnan, R. , & Sudha, V. (2017). Hurdles in brown rice consumption. In A. Manickavasagan , C. Santhakumar , & N. Venkatachakapathy (Eds.), Brown rice (pp. 255–269). Springer International Publishing. 10.1007/978-3-319-59011-0_15

[risa70008-bib-0072] Mojica, L. E. , & Reforma, M. G. (2010). An exploratory market study of brown rice as a health food in the Phillippines. Journal of Agricultural Science, Tokyo University of Agriculture, 55(1), 19–30.

[risa70008-bib-0073] Moore, K. L. , Lombi, E. , Zhao, F.‐J. , & Grovenor, C. R. M. (2012). Elemental imaging at the nanoscale: NanoSIMS and complementary techniques for element localisation in plants. Analytical and Bioanalytical Chemistry, 402(10), 3263–3273. 10.1007/s00216-011-5484-3 22052155

[risa70008-bib-0074] Murray, C. J. L. , Vos, T. , Lozano, R. , Naghavi, M. , Flaxman, A. D. , Michaud, C. , Ezzati, M. , Shibuya, K. , Salomon, J. A. , Abdalla, S. , Aboyans, V. , Abraham, J. , Ackerman, I. , Aggarwal, R. , Ahn, S. Y. , Ali, M. K. , AlMazroa, M. A. , Alvarado, M. , Anderson, H. R. , … Lopez, A. D. (2012). Disability‐adjusted life years (DALYs) for 291 diseases and injuries in 21 regions, 1990–2010: A systematic analysis for the Global Burden of Disease Study 2010. The Lancet, 380(9859), 2197–2223. 10.1016/S0140-6736(12)61689-4 23245608

[risa70008-bib-0075] Naito, S. , Matsumoto, E. , Shindoh, K. , & Nishimura, T. (2015). Effects of polishing, cooking, and storing on total arsenic and arsenic species concentrations in rice cultivated in Japan. Food Chemistry, 168, 294–301. 10.1016/j.foodchem.2014.07.060 25172713

[risa70008-bib-0076] Narukawa, T. , Hioki, A. , & Chiba, K. (2012). Speciation and monitoring test for inorganic arsenic in white rice flour. Journal of Agricultural and Food Chemistry, 60(4), 1122–1127. 10.1021/jf204240p 22224477

[risa70008-bib-0077] Narukawa, T. , Matsumoto, E. , Nishimura, T. , & Hioki, A. (2014). Determination of sixteen elements and arsenic species in brown, polished and milled rice. Analytical Sciences, 30(2), 245–250. 10.2116/analsci.30.245 24521911

[risa70008-bib-0078] Nishimura, T. , Hamano‐Nagaoka, M. , Sakakibara, N. , Abe, T. , Maekawa, Y. , & Maitani, T. (2010). Determination method for total arsenic and partial‐digestion method with nitric acid for inorganic arsenic speciation in several varieties of rice. Food Hygiene and Safety Science, 51(4), 178–181. 10.3358/shokueishi.51.178 20827054

[risa70008-bib-0079] Norton, G. J. , Adomako, E. E. , Deacon, C. M. , Carey, A. M. , Price, A. H. , & Meharg, A. A. (2013). Effect of organic matter amendment, arsenic amendment and water management regime on rice grain arsenic species. Environmental Pollution, 177, 38–47. 10.1016/j.envpol.2013.01.049 23466730

[risa70008-bib-0080] Oberoi, S. , Barchowsky, A. , & Wu, F. (2014). The global burden of disease for skin, lung, and bladder cancer caused by arsenic in food. Cancer Epidemiology, Biomarkers & Prevention, 23(7), 1187–1194. 10.1158/1055-9965.EPI-13-1317 PMC4082465 24793955

[risa70008-bib-0081] Panthri, M. , & Gupta, M. (2022). An insight into the act of iron to impede arsenic toxicity in paddy agro‐system. Journal of Environmental Management, 316, 115289. 10.1016/j.jenvman.2022.115289 35598452

[risa70008-bib-0082] Pasias, I. N. , Thomaidis, N. S. , & Piperaki, E. A. (2013). Determination of total arsenic, total inorganic arsenic and inorganic arsenic species in rice and rice flour by electrothermal atomic absorption spectrometry. Microchemical Journal, 108, 1–6. 10.1016/j.microc.2012.11.008

[risa70008-bib-0083] Pedron, T. , Segura, F. R. , Paniz, F. P. , de Moura Souza, F. , dos Santos, M. C. , de Magalhães Júnior, A. M. , & Batista, B. L. (2019). Mitigation of arsenic in rice grains by polishing and washing: Evidencing the benefit and the cost. Journal of Cereal Science, 87, 52–58. 10.1016/j.jcs.2019.03.003

[risa70008-bib-0084] Pizarro, I. , Gómez, M. , Palacios, M. A. , & Cámara, C. (2003). Evaluation of stability of arsenic species in rice. Analytical and Bioanalytical Chemistry, 376, 102–109. 12734624 10.1007/s00216-003-1870-9

[risa70008-bib-0085] Rahman, M. A. , & Hasegawa, H. (2011). High levels of inorganic arsenic in rice in areas where arsenic‐contaminated water is used for irrigation and cooking. Science of The Total Environment, 409(22), 4645–4655. 10.1016/j.scitotenv.2011.07.068 21899878

[risa70008-bib-0086] Rahman, M. A. , Hasegawa, H. , Rahman, M. A. , Rahman, M. M. , & Miah, M. A. M. (2006). Influence of cooking method on arsenic retention in cooked rice related to dietary exposure. Science of The Total Environment, 370(1), 51–60. 10.1016/j.scitotenv.2006.05.018 16839594

[risa70008-bib-0087] Rahman, M. A. , Hasegawa, H. , Rahman, M. M. , Rahman, M. A. , & Miah, M. A. M. (2007). Accumulation of arsenic in tissues of rice plant (*Oryza sativa* L.) and its distribution in fractions of rice grain. Chemosphere, 69(6), 942–948. 10.1016/j.chemosphere.2007.05.044 17599387

[risa70008-bib-0088] Rahman, M. A. , Rahman, M. M. , Reichman, S. M. , Lim, R. P. , & Naidu, R. (2014). Arsenic speciation in Australian‐grown and imported rice on sale in Australia: Implications for human health risk. Journal of Agricultural and Food Chemistry, 62(25), 6016–6024. 10.1021/jf501077w 24892387

[risa70008-bib-0089] Rathna Priya, T. S. , Eliazer Nelson, A. R. L. , Ravichandran, K. , & Antony, U. (2019). Nutritional and functional properties of coloured rice varieties of South India: A review. Journal of Ethnic Foods, 6(11), 1–11. 10.1186/s42779-019-0017-3

[risa70008-bib-0090] Reid, M. S. , Hoy, K. S. , Schofield, J. R. M. , Uppal, J. S. , Lin, Y. , Lu, X. , Peng, H. , & Le, X. C. (2020). Arsenic speciation analysis: A review with an emphasis on chromatographic separations. TrAC Trends in Analytical Chemistry, 123, 115770.

[risa70008-bib-0091] Ren, X.‐L. , Liu, Q.‐L. , Wu, D.‐X. , & Qing‐Yao, S. (2006). Variations in concentration and distribution of health‐related elements affected by environmental and genotypic differences in rice grains. Rice Science, 13(3), 170–178. http://www.ricesci.cn; http://www.ricescience.org

[risa70008-bib-0092] Romero, M. (2011). Nutritional and health aspects of brown rice. Seminat—Workshop on Maintstreaming Brown Rice to Low‐and Middle‐Income Families.

[risa70008-bib-0093] Roy, P. , Orikasa, T. , Okadome, H. , Nakamura, N. , & Shiina, T. (2011). Processing conditions, rice properties, health and environment. International Journal of Environmental Research and Public Health, 8(6), 1957–1976. 10.3390/ijerph8061957 21776212 PMC3138007

[risa70008-bib-0094] Ruangwises, S. , Saipan, P. , Tengjaroenkul, B. , & Ruangwises, N. (2012). Total and inorganic arsenic in rice and rice bran purchased in Thailand. Journal of Food Protection, 75(4), 771–774. 10.4315/0362-028X.JFP-11-494 22488070

[risa70008-bib-0095] Saleh, A. S. M. , Wang, P. , Wang, N. , Yang, L. , & Xiao, Z. (2019). Brown rice versus white rice: Nutritional quality, potential health benefits, development of food products, and preservation technologies. Comprehensive Reviews in Food Science and Food Safety, 18, 1070–1096. 10.1111/1541-4337.12449 33336992

[risa70008-bib-0096] Sauvé, S. (2014). Time to revisit arsenic regulations: Comparing drinking water and rice. BMC Public Health, 14(1), 465. 10.1186/1471-2458-14-465 24884827 PMC4049411

[risa70008-bib-0097] Schmidt, C. (2015). In search of “Just Right”: The challenge of regulating arsenic in rice. Environmental Health Perspectives, 123(1), A16–A19. 10.1289/ehp.123-A16 25561606 PMC4286262

[risa70008-bib-0098] Schoof, R. A. , Yost, L. J. , Eickhoff, J. , Crecelius, E. A. , Cragin, D. W. , Meacher, D. M. , & Menzel, D. B. (1999). A market basket survey of inorganic arsenic in food. Food and Chemical Toxicology, 37(8), 839–846. 10.1016/S0278-6915(99)00073-3 10506007

[risa70008-bib-0099] Selvam, S. , Masilamani, P. , Umashankar, P. T. , & Alex Albert, V. (2017). Opportunities and challenges in marketing of brown rice. In A. Manickavasagan , C. Santhakumar , & N. Venkatachalapathy (Eds.), Brown rice (pp. 271–282). Springer International Publishing. 10.1007/978-3-319-59011-0_16

[risa70008-bib-0100] Shimabukuro, M. , Higa, M. , Kinjo, R. , Yamakawa, K. , Tanaka, H. , Kozuka, C. , Yabiku, K. , Taira, S. I. , Sata, M. , & Masuzaki, H. (2014). Effects of the brown rice diet on visceral obesity and endothelial function: The BRAVO study. British Journal of Nutrition, 111(2), 310–320. 10.1017/S0007114513002432 23930929

[risa70008-bib-0101] Signes‐Pastor, A. J. , Carey, M. , Carbonell‐Barrachina, A. A. , Moreno‐Jiménez, E. , Green, A. J. , & Meharg, A. A. (2016). Geographical variation in inorganic arsenic in paddy field samples and commercial rice from the Iberian Peninsula. Food Chemistry, 202, 356–363. 10.1016/j.foodchem.2016.01.117 26920305

[risa70008-bib-0102] Signes‐Pastor, A. J. , Zens, M. S. , Seigne, J. , Schned, A. , & Karagas, M. R. (2019). Rice consumption and incidence of bladder cancer in the United States Population. Epidemiology, 30(2), E5–E7. 10.1097/EDE.0000000000000955 30721170 PMC6368392

[risa70008-bib-0103] Smith, E. , Kempson, I. , Juhasz, A. L. , Weber, J. , Skinner, W. M. , & Gräfe, M. (2009). Localization and speciation of arsenic and trace elements in rice tissues. Chemosphere, 76(4), 529–535. 10.1016/j.chemosphere.2009.03.010 19345396

[risa70008-bib-0104] Sofuoglu, S. C. , Güzelkaya, H. , Akgül, Ö. , Kavcar, P. , Kurucaovalı, F. , & Sofuoglu, A. (2014). Speciated arsenic concentrations, exposure, and associated health risks for rice and bulgur. Food and Chemical Toxicology, 64, 184–191. 10.1016/j.fct.2013.11.029 24296133

[risa70008-bib-0105] Sommella, A. , Deacon, C. , Norton, G. , Pigna, M. , Violante, A. , & Meharg, A. A. (2013). Total arsenic, inorganic arsenic, and other elements concentrations in Italian rice grain varies with origin and type. Environmental Pollution, 181, 38–43. 10.1016/j.envpol.2013.05.045 23810819

[risa70008-bib-0106] Su, L. J. , Chiang, T.‐C. , & O'Connor, S. N. (2023). Arsenic in brown rice: Do the benefits outweigh the risks?. Frontiers in Nutrition, 10, 1–4. 10.3389/fnut.2023.1209574 PMC10375490 37521417

[risa70008-bib-0107] Sudha, V. , Spiegelman, D. , Hong, B. , Malik, V. , Jones, C. , Wedick, N. M. , Hu, F. B. , Willett, W. , Bai, M. R. , Ponnalagu, M. M. , Arumugam, K. , & Mohan, V. (2013). Consumer acceptance and preference study (CAPS) on brown and undermilled Indian rice varieties in Chennai, India. Journal of the American College of Nutrition, 32(1), 50–57. 10.1080/07315724.2013.767672 24015699 PMC3769789

[risa70008-bib-0108] Sun, G.‐X. , Williams, P. N. , Carey, A.‐M. , Zhu, Y.‐G. , Deacon, C. , Raab, A. , Feldmann, J. , Islam, R. M. , & Meharg, A. A. (2008). Inorganic arsenic in rice bran and its products are an order of magnitude higher than in bulk grain. Environmental Science & Technology, 42(19), 7542–7546. 10.1021/es801238p 18939599

[risa70008-bib-0109] Sun, Q. , Spiegelman, D. , Van Dam, R. M. , Holmes, M. D. , Malik, V. S. , Willett, W. C. , & Hu, F. B. (2010). White rice, brown rice, and risk of type 2 diabetes in US men and women. Archives of Internal Medicine, 170(11), 961–969. 10.1001/archinternmed.2010.109 20548009 PMC3024208

[risa70008-bib-0110] Suriyagoda, L. D. B. , Dittert, K. , & Lambers, H. (2018). Mechanism of arsenic uptake, translocation and plant resistance to accumulate arsenic in rice grains. Agriculture, Ecosystems and Environment, 253, 23–37. 10.1016/j.agee.2017.10.017

[risa70008-bib-0111] Syu, C. H. , Huang, C. C. , Jiang, P. Y. , Lee, C. H. , & Lee, D. Y. (2015). Arsenic accumulation and speciation in rice grains influenced by arsenic phytotoxicity and rice genotypes grown in arsenic‐elevated paddy soils. Journal of Hazardous Materials, 286, 179–186. 10.1016/j.jhazmat.2014.12.052 25577320

[risa70008-bib-0112] Torres‐Escribano, S. , Leal, M. , Vélez, D. , & Montoro, R. (2008). Total and inorganic arsenic concentrations in rice sold in Spain, effect of cooking, and risk assessments. Environmental Science and Technology, 42(10), 3867–3872. 10.1021/es071516m 18546736

[risa70008-bib-0113] Tsuji, J. S. , Yost, L. J. , Barraj, L. M. , Scrafford, C. G. , & Mink, P. J. (2007). Use of background inorganic arsenic exposures to provide perspective on risk assessment results. Regulatory Toxicology and Pharmacology, 48(1), 59–68. 10.1016/j.yrtph.2007.01.004 17346867

[risa70008-bib-0117] US Environmental Protection Agency/Joint Institute for Food Safety and Applied Nutrition . (2023). What we eat in America‐ food commodity intake database 2005—2010. What we eat in America. EPA/JIFSAN.

[risa70008-bib-0118] Vahter, M. (2008). Health effects of early life exposure to arsenic. Basic & Clinical Pharmacology & Toxicology, 102(2), 204–211. 10.1111/j.1742-7843.2007.00168.x 18226075

[risa70008-bib-0119] Van de Wiele, T. , Laing, G. Du , & Calatayud, M. (2015). Arsenic from food: Biotransformations and risk assessment. Current Opinion in Food Science, 6, 1–6. 10.1016/j.cofs.2015.11.004

[risa70008-bib-0124] WHO . (2008). Guidelines for drinking‐water quality, 3rd edition incorporating the first and second addenda. Vol. 1. Recommendations. World Health Organization. 35417116

[risa70008-bib-0120] WHO, & UNICEF . (2018). Arsenic primer: Guidance on the inestigation & mitigation of arsenic contamination. UNICEF.

[risa70008-bib-0121] Williams, P. N. , Price, A. H. , Raab, A. , Hossain, S. A. , Feldmann, J. , & Meharg, A. A. (2005). Variation in arsenic speciation and concentration in paddy rice related to dietary exposure. Environmental Science and Technology, 39(15), 5531–5540. 10.1021/es0502324 16124284

[risa70008-bib-0122] Williams, P. N. , Raab, A. , Feldmann, J. , & Meharg, A. A. (2007). Market basket survey shows elevated levels of as in South Central US processed rice compared to California: Consequences for human dietary exposure. Environmental Science and Technology, 41(7), 2178–2183. 10.1021/es061489k 17438760

[risa70008-bib-0123] Williams, P. N. , Villada, A. , Deacon, C. , Raab, A. , Figuerola, J. , Green, A. J. , Feldmann, J. , & Meharg, A. A. (2007). Greatly enhanced arsenic shoot assimilation in rice leads to elevated grain levels compared to wheat and barley. Environmental Science and Technology, 41(19), 6854–6859. 10.1021/es070627i 17969706

[risa70008-bib-0125] Wu, F. , Yang, N. , Touré, A. , Jin, Z. , & Xu, X. (2013). Germinated brown rice and its role in human health. Critical Reviews in Food Science and Nutrition, 53(5), 451–463. 10.1080/10408398.2010.542259 23391013

[risa70008-bib-0126] Wu, K. , Gunaratne, A. , Gan, R. , Bao, J. , Corke, H. , & Jiang, F. (2018). Relationships between cooking properties and physicochemical properties in brown and white rice. Starch, 70(5–6), 1700767. 10.1002/star.201700167

[risa70008-bib-0127] Wu, X. , Guo, T. , Luo, F. , & Lin, Q. (2023). Brown rice: A missing nutrient‐rich health food. Food Science and Human Wellness, 12(5), 1458–1470. 10.1016/j.fshw.2023.02.010

[risa70008-bib-0128] Wu, X. , Liu, J. , Li, D. , & Liu, C.‐M. (2016). Rice caryopsis development II: Dynamic changes in the endosperm. Journal of Integrative Plant Biology, 58(9), 786–798. 10.1111/jipb.12488 27449987

[risa70008-bib-0129] Yim, S. R. , Park, G. Y. , Lee, K. W. , Chung, M.‐S. , & Shim, S.‐M. (2017). Determination of total arsenic content and arsenic speciation in different types of rice. Food Science and Biotechnology, 26(1), 293–298. 10.1007/s10068-017-0039-9 30263541 PMC6049464

[risa70008-bib-0130] Yu, J. , Balaji, B. , Tinajero, M. , Jarvis, S. , Khan, T. , Vasudevan, S. , Ranawana, V. , Poobalan, A. , Bhupathiraju, S. , Sun, Q. , Willett, W. , Hu, F. B. , Jenkins, D. J. A. , Mohan, V. , & Malik, V. S. (2022). White rice, brown rice and the risk of type 2 diabetes: A systematic review and meta‐analysis. BMJ Open, 12(9), e065426. 10.1136/bmjopen-2022-065426 PMC9516166 36167362

[risa70008-bib-0131] Zavala, Y. J. , & Duxbury, J. M. (2008). Arsenic in rice: I. Estimating normal levels of total arsenic in rice grain. Environmental Science and Technology, 42(10), 3856–3860. 10.1021/es702747y 18546734

[risa70008-bib-0132] Zavala, Y. J. , Gerads, R. , Gürleyük, H. , & Duxbury, J. M. (2008). Arsenic in rice: II. Arsenic speciation in USA grain and implications for human health. Environmental Science and Technology, 42(10), 3861–3866. 10.1021/es702748q 18546735

[risa70008-bib-0133] Zhao, F. J. , McGrath, S. P. , & Meharg, A. A. (2010). Arsenic as a food chain contaminant: Mechanisms of plant uptake and metabolism and mitigation strategies. Annual Review of Plant Biology, 61, 535–559. 10.1146/annurev-arplant-042809-112152 20192735

[risa70008-bib-0134] Zhao, F.‐J. , Zhu, Y.‐G. , & Meharg, A. A. (2013). Methylated arsenic species in rice: Geographical variation, origin, and uptake mechanisms. Environmental Science & Technology, 47(9), 3957–3966. 10.1021/es304295n 23521218

[risa70008-bib-0135] Zheng, M.‐Z. , Cai, C. , Hu, Y. , Sun, G.‐X. , Williams, P. N. , Cui, H.‐J. , Li, G. , Zhao, F.‐J. , & Zhu, Y.‐G. (2011). Spatial distribution of arsenic and temporal variation of its concentration in rice. New Phytologist, 189(1), 200–209. 10.1111/j.1469-8137.2010.03456.x 20840510

